# Evaluation of Four Lymph Node Classifications for the Prediction of Survival in Hilar Cholangiocarcinoma

**DOI:** 10.1007/s11605-021-05211-x

**Published:** 2022-01-01

**Authors:** Zhi-Peng Liu, Qing-Yi Zhang, Wei-Yue Chen, Yu-Yan Huang, Yan-Qi Zhang, Yi Gong, Yan Jiang, Jie Bai, Zhi-Yu Chen, Hai-Su Dai

**Affiliations:** 1 Department of Hepatobiliary Surgery, Southwest Hospital, Third Military Medical University (Army Medical University), No. 30 Gaotanyan Road, Chongqing, 400038 China; 2 Department of Clinical Center of Oncology, Zhejiang University Lishui Hospital, Lishui, 323000 Zhejiang China; 3 Department of Health Statistics, College of Military Preventive Medicine, Army Medical University, Chongqing, China

**Keywords:** Hilar cholangiocarcinoma, Lymph node metastasis, Lymph node staging system

## Abstract

**Background:**

An important prognostic indicator of hilar cholangiocarcinoma (HCCA) in patients after surgery is metastasis of lymph nodes (LN). However, there are many types of LN staging systems to the issue of a better determination of the prognosis of patients through the lymphatic staging system which needs research. Based on the above, we tried to re-evaluate the staging system of HCCA LNs. We compared the American Joint Committee on Cancer (AJCC), number of metastatic LNs (MLN), ratio of LN (LNR), and log odds of MLNs (LODDS) in individuals undergoing curative resection to determine the best LN staging system.

**Methods:**

In the current study, we retrospectively analyzed 229 patients undergoing curative resection. We evaluated the impact of the stage of AJCC pN, LNR, LODDS, and MLN on OS (overall survival) and RFS (recurrence-free survival). According to the curve of receiver operating characteristic (ROC), we compared the predictive capacity of different staging systems of LN for survival and recurrence.

**Results:**

Multivariate analysis results revealed that LODDS >  − 0.45 (95% CI = 1.115–2.709, *P* = 0.015; 95% CI = 1.187–2.780, *P* = 0.006) are independent risk factors affecting OS and RFS, respectively. Compared with LN status, AJCC pN stage, MLN, and LNR, the variable having the highest area under the ROC curve (AUC) was LODDS when predicting 1-year, 3-year, and 5-year OS and RFS.

**Conclusion:**

This study shows that metastasis of LNs is a key indicator for predicting patient death and recurrence. Among them, LODDS is the best LN staging system for the prognostic evaluation of HCCA patients after surgery. Clinicians can incorporate LODDS into HCCA patient lymphatic staging system for a more accurate prognosis of HCCA patients post-surgery.

**Supplementary Information:**

The online version contains supplementary material available at 10.1007/s11605-021-05211-x.

## Introduction

Hilar cholangiocarcinoma (HCCA) is a highly malignant tumor with an increasing incidence in recent years ^1^. The prognosis of HCCA is usually poor, so an accurate prognosis is extremely important. Metastasis to lymph nodes (LN) is a key tumor prognosis indicator ^2,3^. The prognosis of most tumors is directly related to metastasis of the LN and their number. Studies have shown that more than 30% of patients with HCCA who underwent curative resection have LN metastasis ^4–6^. However, it is still unknown which LN staging system is in use to judge patient prognosis more accurately.

To better describe the status of LNs, some scholars use different methods to classify LN metastases ^7,8^. Among them, the pN stage as per the American Joint Committee on Cancer (AJCC) staging is based on incremental tumor-infiltrated positive LNs ^9,10^. LN ratio (LNR) is defined as the number of positive lymph nodes divided by the total number of lymph nodes examined ^8^. Studies have shown that the LNR is superior to the number of metastatic LNs (MLN) for staging in cholangiocarcinoma ^11–13^. However, LN staging systems such as LNR, MLN, and AJCC pN stage have limitations in predicting the prognosis of patients ^14^. In recent years, the log odds of MLNs (LODDS) has been included by clinicians as a new prognostic indicator ^15^. LODDS were calculated as the log of [MLN** ± **0.5/total number of LNs retrieved (TLN) − MLN + 0.5] ^16^. Kwon et al. have confirmed that LODDS is the best LN staging system for judging the prognosis of patients with ampullary adenocarcinoma ^17^. Zhou et al. used MLN, LNR, LODDS, and other LN staging systems to assess the prognosis of patients with distant metastatic cancer of the stomach and found that LODDS is the most accurate system of LN staging for assessing the prognosis of patients ^18^. Conci et al. evaluated the LN analysis system of 99 HCCA patients and pointed out that LODDS is the best LN staging system for the prediction of the overall survival (OS) time of patients ^19^. However, his study did not compare the performance of the LN staging system in assessing HCCA recurrence.

Thus, this study aimed to compare MLN, LNR, AJCC pN stage values, and LODDS in evaluating the OS and recurrence-free survival (RFS) of patients with HCCA who underwent curative resection and finally determine the most suitable LN staging system.

## Methods

### Patient Selection

We retrospectively evaluated the medical records of 229 newly diagnosed HCCA patients who underwent curative resection in Southwest Hospital of China from January 2006 to December 2019. Each patient was confirmed to be HCCA by postoperative pathology. Patients who were found to be unresectable at exploration, had received adjuvant chemotherapy and radiotherapy, and died within 30 days after surgery were excluded. Curative resection was defined as complete resection of all macroscopic and microscopic HCCA tumor with microscopically clear resection margins in the surgical specimens. Regardless of preoperative computed tomography (CT) or magnetic resonance imaging (MRI) or suspicion of lymph node metastasis, patients underwent loco-regional lymphadenectomy. All lymph node tissue underwent pathological biopsy. This research followed the ethical guidelines of the World Medical Association (WMA; Declaration of Helsinki). The approval for the present research was obtained from the Ethics Committee of Southwest Hospital (approval number KY2021129), and all patients obtained informed, written consent for the clinical study.

### Outcome Measures

The distribution of tumors along the bile duct during our operation is classified as per Bismuth-Corlette ^20^. Classification of the LN location is based on the English version of the 3rd edition of the Japanese Cholangiocarcinoma Classification ^21^. As per the AJCC staging system (8th edition), the LN metastasis involved in the common bile duct, cystic duct, hepatic artery, and portal vein is N1; the metastatic LN involved in the mesentery, abdominal cavity, and para-abdominal aorta is N2 ^9^. LNR is the ratio of the MLN/TLN ^11^. Based on the outcomes of earlier studies, patients were divided into intervals of LNR as LNR0, LNR = 0; LNR1, 0.01 < LNR ≤ 0.25; and LNR2, LNR > 0.25 ^11^. The influence of the number of MLN on the prognosis is ascertained by values for cut-off as 0, 1 ~ 3, and > 3 ^19^. LODDS is defined as log [(MLN + 0.5) divided by (TLN-MLN + 0.5)] ^22^. We defined the intervals of the LODDS LN staging system for HCCA patients according to previous studies but found that the proportion of patients in each interval of LODDS varied remarkably ^19^. Therefore, we performed three equal categorizations according to the numerical order of LODDS. That is, all patients were categorized into the following three LODDS intervals: LODDS1, LODDS ≤  − 0.85; LODDS2, LODDS − 0.85 < LODDS ≤  − 0.45; and LODDS3, LODDS >  − 0.45.

The focus of this study was OS and RFS. OS was defined from the surgery date to the last follow-up or death due to any cause. The definition of RFS was the time from the surgery date to the diagnosis of recurrence or no disease found at the last follow-up. If the patient did not relapse, the definition of RFS was from the surgery date to the last follow-up or the date of death.

### Statistical Analysis

For continuous variables, median and IQR (interquartile range) or the mean and standard deviation were used. Fisher’s exact test or the chi-square test was applied for categorical variables, and Mann–Whitney *U* test was implemented for numerical variables. We used 25 mm and 150 U/L as the cut-off value of tumor size and CA19-9 ^19,23^. The univariate survival rate was determined using the Kaplan–Meier method, and for comparison, the log-rank test was applied. We used the model of Cox regression for multivariate assessments to ascertain independent prognostic criteria. We analyzed the factors having *P* < 0.10 in the univariate analysis as covariates in multivariate analysis. In addition, we calculated risk ratios (HRs) and 95% confidence intervals (CIs), and HR < 1.0 indicates a survival benefit. For an accurate evaluation and comparison of the prognostic ability of different methods of LN staging, we used receiver operating characteristics curve (ROC) and area under the ROC curve (AUC) to compare LODDS, LNR, MLN, AJCC pN stage, LN status, and other indicators in predicting the difference in the mortality and recurrence rate of patients at each time point. Statistical analyses were performed using SPSS® version 26.0 (IBM, Armonk, New York, USA).

## Results

### Basic Clinical Information

The demographic, clinical, and pathological data of 246 HCCA patients who had surgery in the period between January 2006 and December 2019 were collected. Among these patients, 229 patients had complete pathological and clinical data, so they were included in the current survey. A summary of study population data was summarized in Table 1. One hundred fifty-two cases (66.4%) underwent major live resection. All patients underwent loco-regional lymphadenectomy. There were 143 cases (62.4%) in AJCC pN0, 63 cases (27.5%) in AJCC pN1, and 23 cases (10.1%) in AJCC pN2. The median number of LNs examined is 4 (IQR, 1–20); among them, 93 patients (40.6%) took 1–3, 126 patients (55.0%) took 4–10, and 10 patients (4.4%) took more than 10. A total of 86 patients (37.6%) had MLN. The median of MLN was 2 (IQR, 1–9). The MLN of 71 cases (31.0%) was 1–3, and the MLN of 16 cases (6.6%) was > 3. The median of LNR and LODDS were 0.00 (IQR, 0.00–1.00) and − 0.70 (IQR, − 1.52–1.18), respectively.

**Table 1:** Clinical and pathological characteristics of 229 patients with hilar cholangiocarcinoma resected with curative intent

Characteristics		Values
Age, years, median (IQR)		58 (49–66)
Size, mm, median (IQR)		3.0 (2.0–3.8)
Preoperative CA19-9, U/L, median (IQR)		186.1 (60.0–459.0)
Gender, *n* (%)	Male	137 (59.8%)
	Female	92 (40.2%)
Type of hepatectomy, *n* (%)	Minor	77 (33.6%)
	Major	152 (66.4%)
Vascular invasion, *n* (%)	No	136 (59.4%)
	Yes	93 (40.6%)
Perineural invasion, *n* (%)	No	147 (64.2%)
	Yes	82 (35.8%)
Differentiation, *n* (%)	Well-moderated	40 (17.5%)
	Poor	189 (82.5%)
Bismuth type, *n* (%)	I–II	98 (42.8%)
	III–IV	131 (57.2%)
AJCC pN stage, *n* (%)	0	143 (62.4%)
	1	63 (27.5%)
	2	23 (10.1%)
Number LN retrieved, *n* (%)	1–3	93 (40.6%)
	4–10	126 (55.0%)
	> 10	10 (4.4%)
MLN, *n* (%)	0	143 (62.4%)
	1–3	71 (31.0%)
	> 3	15 (6.6%)
LNR, *n* (%)	LNR0 (0)	143 (62.4%)
	LNR1 (0.01–0.25)	29 (12.7%)
	LNR2 (> 0.25)	57 (24.9%)
LODDS, *n* (%)	LODDS1 (≤ − 0.85)	75 (32.8%)
	LODDS2 (− 0.84– − 0.45)	90 (39.3%)
	LODDS3 (> − 0.45)	64 (27.9%)

*IQR*, interquartile range; *LN*, lymph node; *CA19-9*, carbohydrate antigen 19–9; *AJCC*, American Joint Committee on Cancer; *MLN*, number of metastatic LNs; *LNR*, lymph node ratio; *LODDS*, log odds of metastatic lymph node

### Survival Analysis

The OS for 1, 3, and 5 years of the total study population were 73.5%, 32.4%, and 23.0%, respectively, and the median OS time was 23.0 months (95% CI = 19.4–26.6 months). The RFS of 1, 3, and 5 years of the total study population were 54.6%, 25.1%, and 16.8%, respectively, and the median RFS was 16.0 months (95% CI = 13.0–19.0 months).

### Analysis of Risk factors Affecting Survival

The study analyzed the clinical and pathological factors that affected OS. The level of CA19-9 (< 500 vs ≥ 500 U/L, *P* = 0.083), size of tumors (< 2.5 vs ≥ 2.5 cm, *P* = 0.002), vascular invasion (no vs yes, *P* = 0.009), degree of differentiation (medium/well-differentiated compared to poorly differentiated, *P* < 0.001), and LN metastasis (negative vs positive, *P* = 0.010) are the primary risk factors that affect OS in univariate analysis (Table 2).

**Table 2:** Kaplan–Meier analysis of the association between overall survival and clinical and pathological factors in the 229 study patients

Prognostic factors		Kaplan–Meier univariate analysis				
		Median OS (months)	1-year OS	3-year OS	5-year OS	*P*
Age	< 60	23.0	76.8%	37.4%	25.7%	.221
	≥ 60	21.0	69.5%	27.1%	19.8%	
Gender	Male	24.0	74.2%	33.7%	22.9%	.700
	Female	19.0	72.6%	30.7%	23.3%	
CA19-9	< 500 U/L	31.0	78.7%	42.2%	24.4%	.083
	≥ 500 U/L	21.0	70.9%	27.7%	22.1%	
Tumor size	< 25 mm	26.0	78.3%	39.8%	31.1%	.002
	≥ 25 mm	17.0	68.5%	24.7%	14.2%	
Bismuth type	I–II	22.0	74.6%	36.5%	25.3%	.372
	III–IV	24.0	72.2%	27.4%	19.8%	
Type of hepatectomy	Minor	23.0	72.3%	32.0%	21.7%	.875
	Major	22.0	74.2%	32.6%	23.6%	
Vascular invasion	No	25.0	78.4%	38.6%	25.6%	.009
	Yes	16.0	66.5%	22.2%	19.4%	
Perineural invasion	No	24.0	74.0%	34.9%	24.3%	.227
	Yes	18.0	72.8%	27.9%	19.9%	
Differentiation	Well-moderated	25.0	79.2%	36.8%	25.6%	< .001
	Poor	12.0	46.6%	10.8%	10.8%	
LN status	Negative (N0)	25.0	77.4%	38.1%	28.5%	.010
	Positive (N +)	18.0	67.1%	22.6%	12.6%	

*P*, *P* value of the log-rank test

*OS*, overall survival; *CA19-9*, carbohydrate antigen 19–9; *LN*, lymph node

We analyzed the clinical and pathological factors affecting RFS. The level of CA19-9 (< 500 vs ≥ 500 U/L, *P* = 0.092), size of tumor (< 2.5 vs ≥ 2.5 cm, *P* = 0.003), vascular invasion (no vs. yes, *P* = 0.037), degree of differentiation (medium/highly differentiated compared to poorly differentiated, *P* ≤ 0.003), and LN metastasis (negative vs positive, *P* = 0.001) were the primary risk factors affecting RFS in univariate analysis (Table 3).

**Table 3:** Kaplan–Meier analysis and multivariable Cox regression survival analysis of the association between recurrence-free survival and clinical and pathological factors in the 229 study patients

Prognostic factors		Kaplan–Meier univariate analysis				
		Median RFS (months)	1-year RFS	3-year RFS	5-year RFS	*P*
Age	< 60	14.0	52.7%	27.8%	24.8%	.653
	≥ 60	17.0	56.8%	22.4%	10.9%	
Gender	Male	17.0	57.2%	25.1%	17.0%	.590
	Female	13.0	50.7%	25.2%	13.8%	
CA19-9	< 500 U/L	22.0	57.5%	30.3%	20.9%	.092
	≥ 500 U/L	15.0	53.1%	22.7%	14.6%	
Tumor size	< 25 mm	20.0	63.7%	28.7%	20.3%	.003
	≥ 25 mm	10.0	44.9%	21.3%	13.2%	
Bismuth type	I–II	16.0	56.1%	27.2%	20.0%	.434
	III–IV	17.0	52.5%	22.4%	12.9%	
Type of hepatectomy	Minor	18.0	52.6%	23.7%	16.2%	.808
	Major	15.0	55.6%	25.8%	17.3%	
Vascular invasion	No	20.0	59.7%	27.3%	17.5%	.037
	Yes	12.0	47.1%	22.8%	17.3%	
Perineural invasion	No	16.0	54.9%	28.3%	18.7%	.382
	Yes	14.0	54.1%	18.8%	12.9%	
Differentiation	Well-moderated	18.0	59.4%	27.6%	18.1%	.003
	Poor	8.0	31.3%	12.3%	12.3%	
LN status	Negative (N0)	20.0	60.4%	30.9%	20.6%	.001
	Positive (N +)	10.0	44.9%	14.7%	10.7%	

*P*, *P* value of the log-rank test

*RFS*, recurrence-free survival; *CA19-9*, carbohydrate antigen 19–9; *LN*, lymph node

**Fig. 1:**
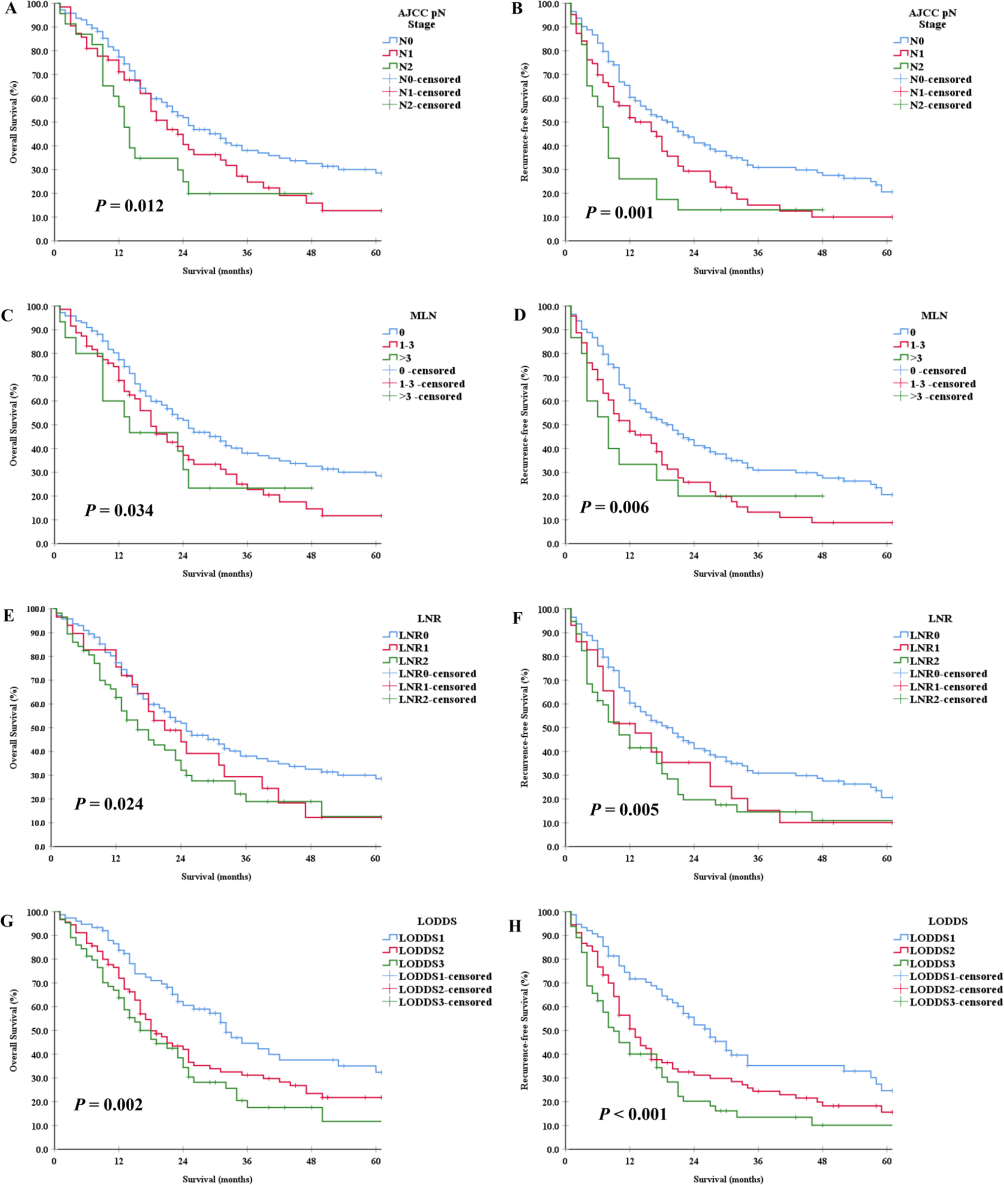
Kaplan–Meier curves of OS and RFS in patients with hilar cholangiocarcinoma underwent surgery by AJCC pN stage (OS, **A**; RFS, **B**), MLN (OS, **C**; RFS, **D**), LNR (OS, **E**; RFS, **F**), and LODDS (OS, **G**; RFS, **H**). OS, overall survival; RFS, recurrence-free survival; AJCC, American Joint Committee on Cancer; MLN, number of metastatic LNs; LNR, lymph node ratio; LODDS, log odds of metastatic lymph node

AJCC pN stage (OS, *P* = 0.012, Fig. 1A; RFS, *P* = 0.001, Fig. 1B), MLN (OS, *P* = 0.034, Fig. 1C; RFS, *P* = 0.002, Fig. 1D), LNR (OS, *P* = 0.024, Fig. 1E; RFS, *P* = 0.005, Fig. 1F), LODDS (OS, *P* = 0.002, Fig. 1G; RFS, *P* < 0.001, Fig. 1H), and other LN staging systems to group HCCA patients. The results indicate that OS and RFS differed significantly in HCCA patients.

The CA19-9 level, vascular invasion, method of resection, tumor size, and degree of differentiation were included as independent variables into the multivariate Cox analysis, and different LN staging methods were used to perform multivariate survival analysis on OS and RFS. Among them, positive LN status (95% CI = 1.125–1.917, *P* = 0.041), AJCC pN2 (95% CI = 1.001–2.940, *P* = 0.049), LNR > 0.25 (95% CI = 1.018–2.250, *P* = 0.040), and LODDS > − 0.45 (95% CI = 1.115–2.709, *P* = 0.015) were independent risk factors affecting OS (Table 4). LN status positive (95% CI = 1.073–2.059, *P* = 0.017), AJCC pN2 (95% CI = 1.138–3.143, *P* = 0.014), MLN 1–3 (95% CI = 1.052–2.078, *P* = 0.024), LNR > 0.25 (95% CI = 1.030–2.200, *P* = 0.035), and LODDS > − 0.45 (95% CI = 1.187–2.780, *P* = 0.006) were independent risk factors affecting RFS (Table 5).

**Table 4:** Kaplan–Meier analysis and multivariable Cox regression survival analysis for overall survival in the 229 patients with hilar cholangiocarcinoma according to each LN staging methods

LN staging methods		Kaplan–Meier univariate analysis					Cox regression analysis	
		Median OS (months)	1-year OS	3-year OS	5-year OS	P	HR* (95% CI)	*P*
LN status	Negative (N0)	25.0	77.4%	38.1%	28.5%	.010	Ref	
	Positive (N +)	18.0	67.1%	22.6%	12.6%		1.363 (1.125–1.917)	.041
AJCC pN stage	0	25.0	77.4%	38.1%	28.5%	.012	Ref	
	1	21.0	71.1%	27.2%	12.7%		1.271 (0.877–1.843)	.206
	2	13.0	56.5%	19.9%	-		1.716 (1.001–2.940)	.049
MLN	0	25.0	77.4%	38.1%	28.5%	.034	Ref	
	1–3	18.0	68.6%	22.7%	11.7%		1.352 (0.948–1.929)	.096
	> 3	14.0	60.0%	23.3%	-		1.430 (0.741–2.760)	.286
LNR	LNR0 (0)	25.0	77.4%	38.1%	28.5%	.024	Ref	
	LNR1 (0.01–0.25)	21.0	75.6%	29.4%	12.2%		1.157 (0.708–1.890)	.561
	LNR2 (> 0.25)	16.0	62.7%	18.9%	12.6%		1.514 (1.018–2.250)	.040
LODDS	LODDS1 (≤ − 0.85)	32.0	83.7%	44.6%	32.4%	.002	Ref	
	LODDS2 (− 0.84– − 0.45)	18.0	72.0%	31.2%	21.8%		1.303 (0.870–1.950)	.199
	LODDS3 (> − 0.45)	16.0	63.7%	17.6%	11.7%		1.738 (1.115–2.709)	.015

* adjusted by carbohydrate antigen 19–9, tumor size, vascular invasion, radicality, and differentiation

*OS*, overall survival; *HR*, hazard ratio; *CI*, confidence interval; *Ref*, reference; *LN*, lymph node; *AJCC*, American Joint Committee on Cancer; *MLN*, number of metastatic LNs; *LNR*, lymph node ratio; *LODDS*, log odds of metastatic lymph node

**Table 5:** Kaplan–Meier analysis and multivariable Cox regression survival analysis for recurrence-free survival in the 229 patients with hilar cholangiocarcinoma according to each LN staging methods

LN staging methods		Kaplan–Meier univariate analysis					Cox regression analysis	
		Median RFS (months)	1-year RFS	3-year RFS	5-year RFS	*P*	HR* (95% CI)	*P*
LN status	Negative (N0)	20.0	60.4%	30.9%	20.6%	.001	Ref	
	Positive (N +)	10.0	44.9%	14.7%	10.7%		1.486 (1.073–2.059)	.017
AJCC pN stage	0	20.0	60.4%	30.9%	20.6%	.001	Ref	
	1	13.0	51.8%	15.0%	10.0%		1.378 (0.966–1.968)	.077
	2	7.0	26.1%	13.0%	-		1.891 (1.138–3.143)	.014
MLN	0	20.0	60.4%	30.9%	20.6%	.006	Ref	
	1–3	12.0	47.3%	13.2%	8.8%		1.478 (1.052–2.078)	.024
	> 3	8.0	33.3%	20.0%	-		1.532 (0.820–2.863)	.181
LNR	LNR0 (0)	20.0	60.4%	30.9%	20.6%	.005	Ref	
	LNR1 (0.01–0.25)	13.0	51.7%	15.2%	10.1%		1.455 (0.913–2.319)	.115
	LNR2 (> 0.25)	10.0	41.5%	14.6%	10.9%		1.505 (1.030–2.200)	.035
LODDS	LODDS1 (≤ − 0.85)	27.0	71.7%	35.2%	24.7%	< .001	Ref	
	LODDS2 (− 0.84– − 0.45)	13.0	50.7%	24.4%	15.6%		1.360 (0.927–1.996)	.116
	LODDS3 (> − 0.45)	9.0	40.1%	13.5%	10.1%		1.817 (1.187–2.780)	.006

* adjusted by carbohydrate antigen 19–9, tumor size, vascular invasion, radicality, and differentiation

*RFS*, recurrence-free survival; *HR*, hazard ratio; *CI*, confidence interval; *Ref*, reference; *LN*, lymph node; *AJCC*, American Joint Committee on Cancer; *MLN*, number of metastatic LNs; *LNR*, lymph node ratio; *LODDS*, log odds of metastatic lymph node

### Comparing the Predictive Ability of the LN Staging System

**Fig. 2:**
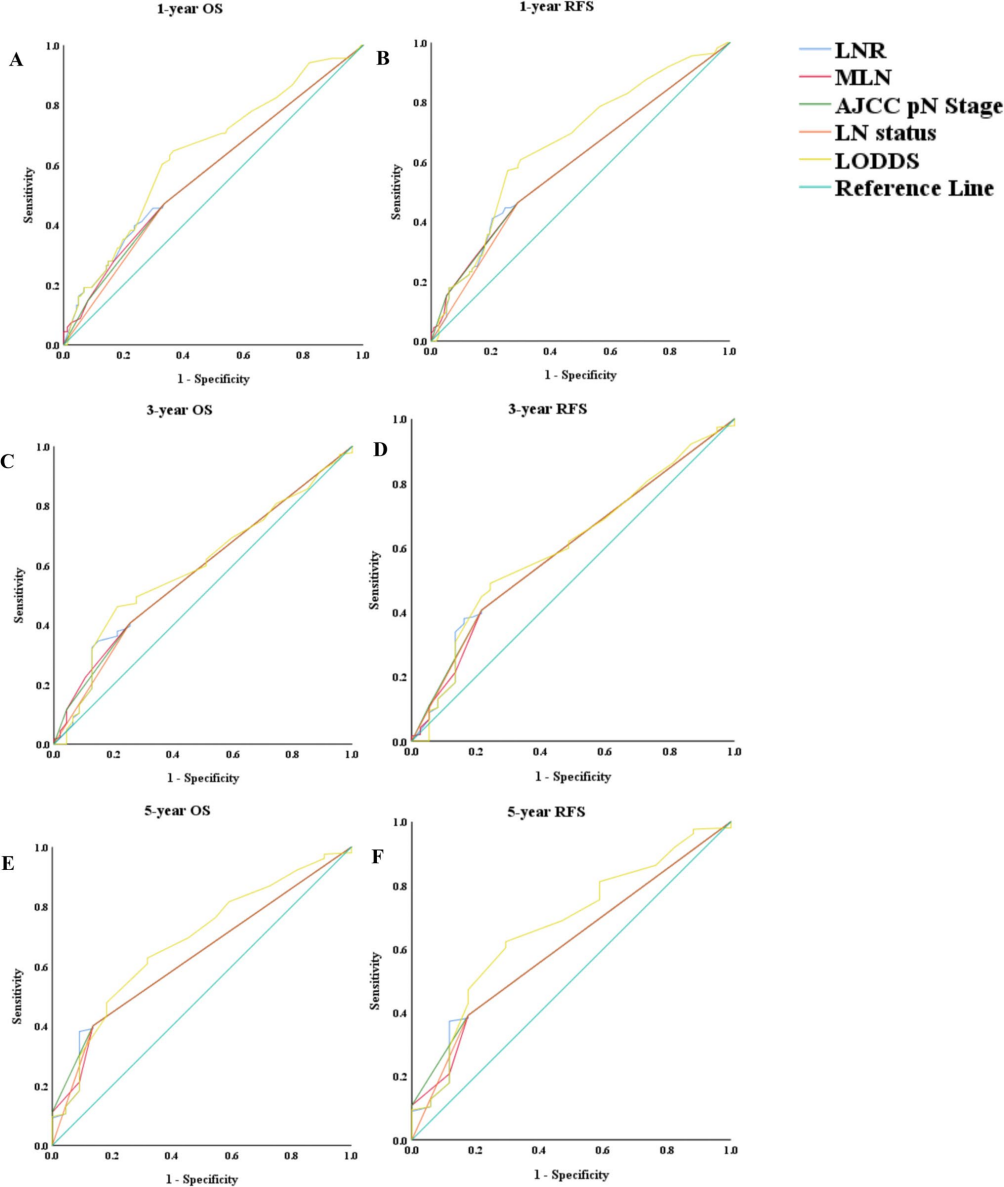
Comparison for the AUC of LODDS, LNR, MLN, AJCC pN stage, and LN status according to 1-year OS (**A**), 3-year OS (**C**), 5-year OS (**E**), 1-year RFS (**B**), 3-year RFS (**D**), and 5-year RFS (**F**). AUC, area under the curve; OS, overall survival; RFS, recurrence-free survival; LN, lymph node; AJCC, American Joint Committee on Cancer; MLN, number of metastatic LNs; LNR, lymph node ratio; LODDS, log odds of metastatic lymph node

Figure 2A, C, and E, respectively, report the cross-validation ROC curve analysis results of various methods of LN staging for OS 1, 3, and 5 years after surgery. In 1-year OS, the variable having the highest AUC was LODDS (AUC = 0.640, 95% CI = 0.561–0.719, *P* = 0.001). In 3-year OS, the variable having the highest AUC was LODDS (AUC = 0.595, 95% CI = 0.508–0.682,*P* = 0.045). In 5-year OS, the variable having the highest AUC was LODDS (AUC = 0.683, 95% CI = 0.571–0.794, *P* = 0.005). Figure 2B, D, and F, respectively, report the cross-validation analysis of the ROC curve of different methods of LN staging for RFS 1, 3, and 5 years after surgery. In 1-year RFS, the variable having the highest AUC was LODDS (AUC = 0.663, 95% CI = 0.539–0.734, *P*** ≤ **0.001). In 3-year RFS, the variable having the highest AUC was LODDS (AUC = 0.603, 95% CI = 0.506–0.690, *P* = 0.047). In 5-year RFS, the variable having the highest AUC was LODDS (AUC = 0.671, 95% CI = 0.542–0.799, *P* = 0.019). The AUC of LODDS, LNR, MLN, AJCC pN stage, and LN status at 1-year, 3-year, and 5-year time point was in Supplement Table 1.

In 1-year OS, the cut-off value of LODDS is − 0.667896. In 3-year OS, the cut-off value of LODDS was − 0.520696. In 5-year OS, the cut-off value of LODDS was − 0.823365. In 1-year RFS, the cut-off value of LODDS was − 0.520696. In 3-year RFS, the cut-off value of LODDS was − 0.667896. In 5-year RFS, the cut-off value of LODDS was − 0.823365.

## Discussion

HCCA is the most common type of cholangiocarcinoma. Currently, surgical resection is the only curable method. LN metastasis has been shown to be a key factor in evaluating the prognosis of patients with cholangiocarcinoma undergoing radical surgery ^24,25^. The AJCC LN staging, 8th edition, is defined according to the number of LN metastasis areas, without considering the LN metastases number. Many studies believe that to assess cholangiocarcinoma prognosis based on the number of LN metastasis areas is controversial ^26,27^. Studies have pointed out that to evaluating the location of LN metastasis is not sufficient. The number of LN metastases also has a direct impact on the prognosis of patients ^28^. In this study, we also got similar results. We found that in assessing patients’ prognoses, univariate results suggest that AJCC LN staging pN0, pN1, pN2 HCCA patients, their postoperative OS, and RFS were statistically different (OS, *P* = 0.012; RFS, *P* = 0.001). HCCA patients with MLN 0, 1–3, and > 3 also had statistical differences in their postoperative OS (*P* = 0.034) and RFS (*P* = 0.002). The results of this study believe that the location and number of LN metastases can affect patients’ prognosis. To further explore the effect of the number of LN metastases on the prognosis, we tried to use more methods according to the LN metastases number to evaluate the patient’s OS and RFS, such as LNR and LODDS, to estimate the best LN staging system for evaluating the patient’s prognosis.

Many scholars believe that the sufficient LN examination number is the prerequisite for the accurate number of positive LNs in gastrointestinal tumors ^29–33^. A large-scale study collected the clinicopathological data of 20,068 patients with gallbladder cancer, ampullary cancer, and extrahepatic bile ducts from the SEER Cancer Registry. More than 10 LN examinations are needed to accurately stage the LNs ^34^. However, it is controversial that expanding the scope of lymph node dissection is beneficial to patients with HCCA. Hakeem et al. indicated that the prognosis of HCCA patients with more than 20 lymphatic examinations was worse ^6^. They believed that more lymph node examinations may not improve the prognosis of patients with hilar cholangiocarcinoma after surgery. The more LNs checked, the more traumatic the operation was. Our team believes that the most suitable system for LN staging should be found in the limited number of LN examinations. Moreover, it should be accurate to assess the prognosis of patients and avoid deliberately taking more LNs for examination. The median of the number of LN examinations in this study was only 3 (range, 1–9).

The number of LNs that were positive increases as the number of LN examinations increases. Therefore, it is inaccurate to estimate the prognosis based alone on the number of positive LNs. LODDS and LNR are the systems of LN staging according to the number of positive LNs proposed in recent years. As per various studies, LODDS and LNR are more reliable in assessing the prognosis of gastrointestinal tumors ^11,13,15,16,35–37^.

In the current study, the number of positive LNs, AJCC LN staging, LODDS, and LNR were used to assess the OS of HCCA patients who underwent radical surgery. Our results indicate that the AUC of LODDS was significantly higher than other LN staging systems when evaluating patients with 1-, 3-, and 5-year OS. This also suggests that LODDS was found to be the best LN staging system for predicting patient mortality.

At present, studies have pointed out that LODDS has better predictive performance in the assessment of gastrointestinal cancer. Zhou et al. analyzed the clinicopathological data of 1999 patients retrospectively with distant metastatic gastric cancer and pointed out that when predicting 1-year cancer-specific survival (CSS) and 2-year CSS, compared with MLN and LNR, LODDS had the highest AUC ^18^. In addition, there are also studies comparing different LN analysis systems in evaluating the prognosis of HCCA patients undergoing radical surgery. Conci et al. compared the predictive capacity of LODDS, MLN, and LNR on OS in 99 HCCA patients. They believe that LODDS has the highest AUC when predicting 3-year OS ^19^. Therefore, Conci believes that LODDS is the best LN staging system when predicting patient mortality. After analyzing the clinicopathological data of 437 HCCA patients, Bagante et al. concluded that a minimum of four LNs should be assessed for HCCA patients undergoing radical surgery ^38^. When the number of ELNs is greater than 4, LODDS shows better prediction performance than the AJCC pN stage for evaluating OS ^38^. However, none of the above reports on the prognostic relationship between LODDS and HCCA mentioned the relationship between the system of LN staging and tumor recurrence. We hypothesize that if a different LN staging system is used to evaluate the tumor recurrence of HCCA patients, that is, to evaluate the patient’s RFS, whether the same conclusions as the evaluation of OS can be obtained, that is, LODDS is the best LN staging system for preciting the mortality rate of HCCA. Based on this, we used LODDS, LNR, the number of positive LNs, and AJCC LN staging to evaluate the 1-, 3-, and 5-years RFS of HCCA patients and found that the AUC of LODDS was the highest. Therefore, we conclude that LODDS is the best LN staging system for predicting the recurrence rate of HCCA patients.

This study has the following limitations. Due to the retrospective nature, all collected data were biased. This study was like other earlier reported studies conducted at single centers. In addition, the pathological results of each case in this study were examined by the different hepatobiliary pathology team while using the same pathological examination process. At the same time, our study lacked external verification. Some variables that are considered critical to predicting prognosis were missing, such as comorbidity, microvascular invasion, or patients’ performance status. However, the results of the current study can improve the debatable issue of the LN staging system in HCCA.

## Conclusions

In summary, LN metastasis is a key factor in judging prognosis. LODDS, on the basis of number of positive LNs, seems to be the best LN staging system for predicting the mortality and recurrence rate of HCCA patients after radical resection. In addition, proper LN dissection is of great significance for clarifying tumor staging and selecting treatment methods.

## Supplementary Information

Below is the link to the electronic supplementary material.

Supplementary file1 (DOCX 21 KB)

## Abbreviations

IQR: Interquartile range
OS: Overall survival
RFS: Recurrence-free survival
AUC: Area under the curve
HR: Hazard ratio
CI: Confidence interval
Ref: Reference
LN: Lymph node
CA19-9: Carbohydrate antigen 19–9
AJCC: American Joint Committee on Cancer
MLN: Number of metastatic LNs
LNR: Lymph node ratio
LODDS: Log odds of metastatic lymph node
