# Supplementary material for: Evaluation of Four Lymph Node Classifications for the Prediction of Survival in Hilar Cholangiocarcinoma
Source: J Gastrointest Surg. 2022 Jan 1;26(5):1030–40. doi: 10.1007/s11605-021-05211-x (PMC9085675; doi:10.1007/s11605-021-05211-x)
Supplement: Supplementary file 1 — Supplementary file1 (DOCX 21 KB) [file 11605_2021_5211_MOESM1_ESM.docx]

**Supplement Table 1**. The AUC of LODDS, LNR, MLN, AJCC pN St. and LN status at 1-year, 3-year and 5-year time point

| Survival | Time point | LODDS | | LNR | | MLN | | AJCC pN St. | | | LN status | |
| --- | --- | --- | --- | --- | --- | --- | --- | --- | --- | --- | --- | --- |
|  |  | AUC | 95%CI | AUC | 95%CI | AUC | 95%CI | AUC | 95%CI | AUC | | 95%CI |
| OS | 1-year | 0.640 | 0.561-0.719 | 0.584 | 0.501-0.668 | 0.577 | 0.494-0.660 | 0.574 | 0.491-0.657 | 0.432 | | 0.350-0.515.3 |
|  | 3-year | 0.595 | 0.508-0.682 | 0.581 | 0.493-0.669 | 0.583 | 0.497-0.570 | 0.581 | 0.494-0.668 | 0.424 | | 0.336-0.513 |
|  | 5-year | 0.683 | 0.571-0.794 | 0.634 | 0.526-0.742 | 0.634 | 0.526-0.741 | 0.640 | 0.536-0.743 | 0.368 | | 0.259-0.477 |
| RFS | 1-year | 0.663 | 0.539-0.734 | 0.596 | 0.523-0.670 | 0.597 | 0.523-0.671 | 0.597 | 0.523-0.671 | 0.587 | | 0.513-0.661 |
|  | 3-year | 0.603 | 0.506-0.690 | 0.594 | 0.499-0.690 | 0.591 | 0.495-0.687 | 0.596 | 0.501-0.690 | 0.595 | | 0.500-0.690 |
|  | 5-year | 0.671 | 0.542-0.799 | 0.610 | 0.484-0.736 | 0.609 | 0.484-0.735 | 0.617 | 0.497-0.737 | 0.608 | | 0.480-0.735 |

**Abbreviations:** AUC, area under the curve; OS, overall survival; RFS, recurrence-free survival; CI, confidence interval; LN, lymph node; AJCC, American joint committee on cancer; MLN, number of metastatic LNs; LNR, lymph node ratio; LODDS, log odds of metastatic lymph node.
